# Bioethics education in clinical settings: theory and practice of the dilemma method of moral case deliberation

**DOI:** 10.1186/s12910-016-0125-1

**Published:** 2016-07-22

**Authors:** Margreet Stolper, Bert Molewijk, Guy Widdershoven

**Affiliations:** Department of Medical Humanities, EMGO+ Institute for Health and Care Research, VU University medical centre (VUmc), De Boelenlaan 1089a, 1081 HV Amsterdam, The Netherlands; Centre of Medical Ethics, University of Oslo, Oslo, Norway

**Keywords:** Education, Clinical setting, Moral Case Deliberation, Dilemma method, Moral competence

## Abstract

**Background:**

Moral Case Deliberation is a specific form of bioethics education fostering professionals’ moral competence in order to deal with their moral questions. So far, few studies focus in detail on Moral Case Deliberation methodologies and their didactic principles. The dilemma method is a structured and frequently used method in Moral Case Deliberation that stimulates methodological reflection and reasoning through a systematic dialogue on an ethical issue experienced in practice.

**Methods:**

In this paper we present a case-study of a Moral Case Deliberation with the dilemma method in a health care institution for people with an intellectual disability, describing the theoretical background and the practical application of the dilemma method. The dilemma method focuses on moral experiences of participants concerning a concrete dilemma in practice. By an in-depth description of each of the steps of the deliberation process, we elucidate the educational value and didactics of this specific method.

**Results:**

The didactics and methodical steps of the dilemma method both supported and structured the dialogical reflection process of the participants. The process shows that the participants learned to recognize the moral dimension of the issue at stake and were able to distinguish various perspectives and reasons in a systematic manner. The facilitator played an important role in the learning process of the participants, by assisting them in focusing on and exploring moral aspects of the case.

**Discussion:**

The reflection and learning process, experienced by the participants, shows competency-based characteristics. The role of the facilitator is that of a Socratic teacher with specific knowledge and skills, fostering reflection, inquiry and dialogue.

**Conclusion:**

The specific didactics of the dilemma method is well suited for teaching bioethics in clinical settings. The dilemma method follows an inductive learning approach through a dialogical moral inquiry in which participants develop not only knowledge but also skills, attitude and character. The role of a trained facilitator and a specific view on teaching and practicing ethics are essential when using the dilemma method in teaching health care professionals how to reflect on their own moral issues in practice.

## Background

Bioethics education is mostly developed and performed in the academic context, for example in bachelor and master programs in medical education or nursing education [1–3]. Usually concepts and theoretical frameworks from ethics are presented via didactical measures such as reading and lecturing [4–6]. Often, hypothetical cases are discussed, using one of the theoretical frameworks from the ethics literature. This approach aims to foster knowledge about theoretical concepts, theories and methods of moral reasoning to students. Using hypothetical cases, to be analysed deductively with theoretical concepts and principles, is important. At the same time it has several limitations when it comes down to bioethics teaching in healthcare practice. One of the limitations is that the focus on cognitive knowledge transfer tends to result in neglecting the importance of skills, attitude and character development. Another limitation is that the moral questions and the normative framework or ethical principles are determined beforehand, without providing room to moral question and principles emerging from the case and the professionals involved. A third limitation is that ethical expertise and insights from *outside* the context are being *applied to* the unique situation, without taking into account sufficiently the experiences and insights of the health care professionals themselves.

In health care practice, actual cases with concrete moral questions are often used for reflection and discussion with the aim to gain answers or solutions. Yet, such discussions tend to lack structure, theoretical depth and attention for moral reasoning, as the focus is on solving the case at hand practically. Moral Case Deliberation (MCD) aims to combine reflection on concrete cases with methodical procedures to foster moral learning [7–9]. In MCD, health care professionals (physicians, nurses, social workers, etc.), but also managers, family and patients, discuss a moral question in a real case within clinical setting. MCD can be regarded as a form of Clinical Ethics Support (CES) in health care, helping health care professionals to reflect systematically on their actual ethical questions and reasoning, and to find answers in acute cases [10, 11]. However, MCD can also be used for teaching bioethics in both educational and clinical settings.

In this paper, we focus on the educational value and didactics of Moral case Deliberation, elaborating the various steps in the dilemma method. The focus is not on the support provided by the Moral Case Deliberation to the professionals, and the assistance in finding a solution to their moral problem, but in the contribution of the dilemma method to a process of joint learning, resulting in knowledge and skills which are relevant for dealing with ethical issues in practice. Moral knowledge addressed in the dilemma method concerns, amongst others, discerning moral topics, values and norms. Skills entail listening and asking the right questions, rather than convincing the other, and being open to other viewpoints, postponing once own judgements [7, 8, 12, 13]. In these learning processes, dialogue is used as a form of moral inquiry in which participants’ insights and conclusions emerge during a process of critical reflection. The ethics expertise of the facilitator requires a theoretical understanding of (the rationale for) dialogue, practical rationality and Socratic epistemology [14–16]. This differs from the ethics expertise of more traditional ethics teachers who tend to focus on transfer of knowledge of ethical concepts and theories.

## Methods

In this article we present a descriptive case-study of the dilemma method in MCD. The case-study does not focus on the case which is investigated during the MCD. The emphasis is on the dilemma method as an intervention. A descriptive case-study is an empirical inquiry that describes and explores an intervention in real life and is particularly suitable for research questions that are focussed on what, why and how the intervention is like it is [17–19]. It is an in-depth study that highlights the particularity, complexity and uniqueness of a single case; the boundaries between the intervention and context are not clearly evident [20]. The approach of a descriptive case study is particularly valuable for developing theory and interventions because of its flexibility and rigor.

The aim of this article is to show an alternative way of teaching bioethics in the clinical setting by describing the theory and practice of the dilemma method as a specific conversation method. This method has been developed and described before [21] and was further developed in the last decade by the authors of this article in cooperation with others. In this article we will describe how this method works in practice, illustrated with a case example. We will discuss the usefulness of the dilemma method for teaching bioethics to professionals in a practical setting, focusing on both the process of learning as experienced by the participants and the role of the facilitator as teacher.

## Theoretical background of MCD

The dilemma method, which is elaborated in this article, is based on a specific view on ethics and moral learning: hermeneutic ethics [22]. It emphasizes practical rationality (*phronèsis*) [23], the importance of dialogue as a way of learning through exchange of perspectives and fusion of horizons [24] and Socratic epistemology [25–27]. A core element of hermeneutic ethics is the central role of actual experiences in daily practice: the validity and reliability of knowledge (claims) and moral judgments are constructed and examined *in* and *with* the practice itself [22]. In the end, the reliability and validity of the judgments gets determined in experience and in the practice of daily life [15]. Moral action is always contextual and temporary; it requires phronesis [6]. Moral knowledge can be enriched by exchanging perspectives in dialogue [22, 28]. Socratic epistemology holds that people have moral knowledge, but need the help of a facilitator (the Socratic midwife) to get access to that knowledge and make it explicit. The art of Socratic questioning can been seen as a tool to foster a critical attitude to examine experiential knowledge and to scrutinize the reasoning processes attached to that.

This does not imply that ethics theories and concepts are not used or are not useful within MCD. The MCD facilitator or the MCD participants themselves can refer to existing theories and concepts, as well as existing normative frameworks (such as policies, laws, professional codes etc.). They can be an important input in order to challenge both the presuppositions and the reasoning within MCD. They can be used for heuristic purposes. However, they are neither used as the starting point nor as the final epistemological and normative arbiter in order to assess the validity and reliability of certain judgments.

The dilemma method focuses on experiences of professionals in practice [29]. Ethical issues are not defined beforehand, but are derived from practice. In MCD, the moral problem under consideration is always a concrete moral issue, experienced by one of the participants. This issue is presented as a case (for example concerning a treatment decision with an individual patient). The case is analysed, not by deductively applying general moral concepts or principles, but by investigating values and norms of the stakeholders in the case. The dilemma method aims to stimulate reflection on *personal* moral experiences and considerations, and the discrepancy among the views and experiences of other participants in the MCD.

Some key principles of MCD that arise from the theories mentioned above are: 1) experience as a starting point for moral reflection; 2) take into account variations related to interpretations and appreciations of facts by the participants of MCD plus the conclusions allied by them; 3) linking the values and norms of the participant to concrete facts in the case; and 4) dialogue as a process and product in which knowledge and practical wisdom emergence and fleshed out by learning by doing [22].

In MCD, participants inquire, with the help of the facilitator, moral questions in *the concrete experience* within the case. A theoretical issue is not a proper starting point for deliberation. Next, MCD emphasizes the variation in thoughts and experiences of health care professionals. In a MCD, different viewpoints are examined and scrutinized. The initial aim is not to decide which perspective or answer is right, but to ask open and critical questions in order to elaborate assumptions behind the perspective, and find out how it is applicable to the case at hand. When one of the participants brings in an ethical notion, for instance the concept of autonomy, the focus will be on examining what autonomy means for this person in this case, and why it is regarded as important. This may result in a deliberation on various interpretations of autonomy, and their relevance for the argumentation with respect to the dilemma in the case. The result of that joint inquiry process is a temporary and context-dependent answer. The insights emerging from an MCD may be valuable in similar new situations, but can never be automatically transposed.

## The dilemma method in practice

In this section we will describe the dilemma method, and show how it works in practice, using a concrete moral case deliberation as an example. We will elaborate the steps, by first presenting the example, and then discussing the aim and procedure of the step under consideration.

### The setting

*A group of 12 health care professionals working in the care for people with an intellectual disability sit together in the living room in one of the houses for sheltered living. The meeting is organized to discuss a problem in the support of one of the clients, Harry. All employees involved in the care for Harry have been invited. Harry is not able to attend the meeting due to his intellectual disability therefore three family members of Harry are present in order to represent both Harry’s and their own view on the case at hand. Two members of the ethical committee of the health care institution participate, one of them as MCD facilitator. Marian will present the case since she is the personal supervisor of Harry. The participants sit in a circle. There is a flipchart available for the facilitator to write down the case, the central moral question and the findings during the various steps within the MCD.*

### Step 1. Introduction

*The facilitator welcomes the participants, especially the family. She explains the issue which will be addressed at the meeting: a problem in the care for Harry. She explains shortly the theoretical background and procedure of the MCD and emphasizes the confidentiality of the meeting. Together with the participants the facilitator formulates the aim of the meeting: elucidating the problem and finding a way of dealing with it.*

During the first step, the aim and procedure of MCD is explained by the facilitator. The facilitator addresses issues such as: what is MCD, what is the aim of this meeting for the participants, what are the mutual expectations (e.g. open and honest communication), and the explanation of the steps in the method. Also the occasion and the context of the MCD are introduced.

The aim of the specific MCD meeting which takes an average of 90 min, is not determined beforehand, but determined by the group. The aim should be kept in mind by the facilitator during the process of deliberation. In case the aim is a decision by one of the participants at the end of the MCD (taking into account the views from others) the facilitator has to take care of the time in order to create space for making a reasoned decision. If the aim is to gain mutual understanding, time for decision-making is not needed. Instead, the focus of the last phase of the meeting will be on elaborating similarities and differences regarding the moral considerations of the participants.

### Step 2. Presentation of the case

*Marian briefly sketches the case: Harry (56 year old) was transferred one year ago from another residence in a village nearby because the sheltered home in which he lived needed to be renovated. He was told that he would return to his former home after the renovation. Harry is doing very well in his new environment. He can work in the garden. He is liked because he often helps other people. In his old residence, he had little to do, and he often was made fun of in the village. Over the past weeks, Harry repeatedly asked when the renovation would be finished, so that he can return. When he brings up the subject, Marian explains to him how well he is doing right now. But Harry keeps insisting that he wants to move back to his old home because that was promised to him. Marian indicates that she does not know what to do, how to respond to Harry’s wish. The facilitator asks Marian at which moment she experienced the problem most strongly. Marian says this was during the last conversation with Harry on this subject three days ago. The facilitator invites Marian to describe this conversation for the other attendees and explain her feelings. She pictures the situation: she met Harry in the garden, he immediately started talking about the renovation, indicating that he wanted to know when he could return. She felt uncertain about what to answer, since the renovation was nearly finished, but she wanted to make Harry understand that a return to his former home would mean that he would no longer have the current opportunities for doing work and helping other people.*

This step focuses on the experience of the case presenter. The presenter is asked to describe a concrete personal situation in which he or she experienced the moral issue at stake. This can be in the past or in the present. As a case can refer to an ongoing process, the presenter is invited to focus on a specific moment within the time line of the case, in which he/she experienced most strongly his/her moral dilemma. This moment is called ‘the moment of heat’ of the case. The case-presenter is asked to provide a short but thick description of the facts of the situation at that moment. Facts include ‘feelings’ he or she experienced since feelings can be useful to better understand the moral discomfort of the presenter and since feelings often implicitly refer to certain values [30].

### Step 3. Formulating the moral question and the dilemma

*The facilitator invites Marian to formulate the moral question, and suggests to the other participants to help Marian in this. The following moral question is formulated: ‘Do we have to do what is promised to Harry?’ Next, the facilitator asks Marion to describe the two alternative actions from which she has to choose. She formulates her dilemma as follows:*

- *A: I follow the wish of Harry and let him move back to his old place.*
- *B: I make Harry stay where he lives now.*

*The facilitator asks the case owner to make a list of the negative consequences of both choices. She notes down on the flip chart:*

- *A: When I follow the wish of Harry and he will go back to his old home, he will have less opportunity to help people and he will risk to be made fun of again.*
- *B: When I make Harry stay where he lives now, I will not respond to his wish and he will continue to repeat the wish.*

In this step, the case-presenter’s underlying moral question is made explicit. By formulating his/her moral question, the other participants can better understand what is at stake and what (morally) matters for the case-presenter.

Often health care professionals struggle with formulating the moral question. At such moments, the MCD facilitator might ask: What is at stake for you in this situation? What worries you? What makes you feel uneasy? Furthermore, to make the moral question more concrete, the case presenter is asked to formulate the situation in terms of a dilemma: what are the concrete actions you could choose for in this situation? In a dilemma, there are always two options which mutually exclude one another. Each of the actions has negative consequences. Formulating explicitly the negative consequences of each of the two options makes clear what is at stake for the case presenter.

### Step 4. Clarification in order to place oneself in the situation of the case presenter

*The facilitator invites all participants to ask questions for clarification concerning the situation. The following questions were asked:*

- *What was the attitude of Harry when he mentioned his wish?*
- *How firm did he express his wish?*
- *Will the old housemates of Harry return to the former home?*
- *What kinds of people make fun of him?*

The fourth step aims to foster a clear understanding of the situation, so that the participants can put themselves in the shoes of the case presenter. The aim of clarification is to (re)construct as clearly as possible the situation presented by the case owner in order to investigate the moral dilemma. Hence, not all facts and all clarification questions are relevant; only those related to the dilemma. All participants put themselves as much as possible in the position of the case-presenter at the moment of heat. This is important because all the participants later will be invited to answer the dilemma question of the case presenter for themselves: how would I answer the moral question if I were in Marian’s situation and how do I justify my own answer? Within MCD, participants try to answer the dilemma of the case presenter. In this meeting, Marian asks what to do. So the participants have to ask what they think they themselves should do in such a situation. Of course, in this case, Marian’s final choice for action A or B is indirectly related to the question how Marian conceives the best interest of Harry, and also how Harry thinks about that. That is important as well, and will get attention in the next step. The clarification in this step does not aim at getting insight into what other people, such as Harry, think, but to better understand the dilemma experienced by Marian, and to prepare the participants to put themselves in her shoes.

### Step 5. Analysing the case in terms of perspectives, values and norms

*The facilitator asks the participants to make explicit the values of the various stakeholders in the case, related to the dilemma. For each value, the group is also invited formulate a normative rule of action (a norm) which follows from the value. She notes down the results systematically on the flip chart.*

**Table:** 

*Perspectives*	*Values*	*Norm*
*Marian (personal supervisor)*	*Happiness* *Being consequent* *Honesty*	*I have to foster Harry’s happiness* *A promise should be kept* *I should tell Harry when the renovation of the house is finished*
*Frederic (team member)*	*Autonomy* *Well being*	*I/we (as a team) have to respect Harry’s wish* *We must take care for Harry’s development*
*Lizz (team leader)*	*Autonomy* *Participation*	*We should follow Harry’s wish* *We should foster Harry’s contribution to social life*
*Harry*	*Independency* *Helpfulness*	*I must take part in decisions about my place of living* *I want to help others*
*Family*	*Involvement* *Protection* *Self esteem*	*We need to keep an eye on Harry* *We must ensure that Harry does not get into trouble* *Harry ought to be not harassed*
*The health care institution*	*Self-determination* *Support* *Involving social network*	*Our clients should decide about their lives* *We should support the clients in realizing their personal goals* *We should actively involve the family in the care for Harry*

To gain insight in the complexity of the case, the participants investigate the values and norms of the stakeholders involved, and jointly construct a perspectives, values, and norms diagram. That means that the participants make a list of the relevant perspectives (stakeholders), and for each perspective investigate what are important values related to the dilemma, and what action should be done to realize a specific value (this we call a norm). In case the group seems to overlook an important stakeholder, value or norm, the facilitator can verify this by asking questions to the participants. For example, “do you think that X would be a relevant perspective which should be included?”. With respect to the representation of a perspective that is not actually attending the MCD session, the MCD facilitator needs to challenge the participants by asking whether they think the absent perspective is represented well enough. The facilitator or the group can also suggest to plan additional actions after the MCD session in order to check (again) whether the norms and values discussed here (still) fit with the absent perspective, especially when the representation plays a central role in the final answer of the moral question (as is the case in this example with Harry). Obviously, in situations when we deal with representing persons with a severe mental handicap or disorder (e.g. dementia) this can be challenging.

The analysis of the perspective of the case presenter will entail values and norms which either support choice A or choice B. Not all stakeholders need to have values and norms which go in both directions. Some will have a clear preference for one of the options, and experience no dilemma themselves. Only values and norms related to the dilemma or moral question are relevant here. The values and norms are not formulated in general; they are always related to a perspective, and expressed in the way they are concretely experienced by the stakeholder under consideration. Thus, the values are not derived from moral theory, but from lived experience.

### Step 6. Looking for alternatives

*The facilitator asks the participants to formulate alternative actions. What other options can be thought of besides making Harry return to his former home or having him stay where he lives now?*

*Various options are suggested:*

- *Make Harry live with his family*
- *Try a return for one month, and evaluate*
- *Do not address the issue anymore*

The aim of this step is to have a brainstorm in order to get a view on possible courses or actions which lie beyond the dilemma. The focus is on stimulating creative out-of-the-box thinking (not on the desirability or feasibility of the alternatives). Some of the alternatives mentioned might be useful later, when participants answer the moral dilemma question for themselves and reflect on their underlying considerations.

### Step 7. Making an individual choice and making explicit one’s considerations

*The facilitator asks the participants to take pen and paper and individually answer the following questions:*

- *It is morally justified that I choose option … (A, B or an alternative).*
- *Because of…. (which value or norm?)*
- *Despite of…. (which value or norm?)*
- *How can you limit the damage of your choice mentioned under c?*
- *What do you need to act according your answer under ‘a’?*

*The facilitator asks who has chosen option A. Carleen, the physiotherapist of Harry and a team member, says her choice is option A and reads out what she wrote down:*

- *I think it is morally justified to act in line with option A (moving back)*
- *Because of Harry’s self-determination*
- *Despite of Harry’s happiness*
- *I would intensify support and try to foster social participation in the village (I would visit meetings together with him et cetera).*
- *I need support and agreement of the family. I also need financial means to deploy more staff.*

*Next the facilitator asks who chose for option B. John, a team member and caregiver of Harry, answers first and reads out:*

- *To me it is morally justified to act in line with option B (not moving back)*
- *Because of Harry’s happiness and my responsibility; I am employed here and it is my duty to make the clients happy instead of unhappy.*
- *Despite of hurting Harry’s trust in us (breaking a promise)*
- *I would admit not being able to keep the promise and meet Harry’s wish in another way, for example by going with him to the garden fair.*
- *I need the support of all team members; we should all choose the same option.*

*The facilitator asks the other participants what choice they made and what considerations can be add to those of Carleen and John. The facilitator notes down all the answers on the flip chart.*

The aim of this step is to have the participants formulate their personal views, values and norms in relation to the case. The moral justification they give is a personal viewpoint on what is morally right, including the moral arguments why they conceive this specific answer or action as morally right. Later on in the MCD process, every moral justification of the MCD participants can be further explored and criticized in order to further learn from each other’s reasoning. The aim of this step is not to give advice to the case-presenter (‘you should do this’), but to examine one’s own thinking concerning the central moral question in the case. The participants chose between option A and B, or an alternative (either mentioned in step 5, or not) including the main value or norm that motivates their choice. Here, referrals to existing normative frameworks (like policy’s, laws or professional codes) can also be mentioned. Furthermore, each of them reflects on the value and norm which cannot be realized, but is still important, and in need of repair. Each participant also makes explicit what he or she needs to repair the so-called ‘moral damage’ which is often an inherent feature of a moral dilemma.

### Step 8. Dialogical inquiry

*Most of the participants have chosen option B, not making Harry return. The values mentioned are compared. Some, like John, consider ‘happiness of Harry’ the main value. Others have mentioned ‘participation in society’. The family members all have chosen option B. For them it is important that Harry is protected against been bullied.*

*The facilitator asks what value is under pressure for those who have chosen option B. For most participants this is ‘self-determination’. This value motivated Carleen to choose option A. The facilitator asks Carleen to elucidate her understanding of self-determination in this specific situation. Carleen says that to her self-determination means people should be able to make choices, even if these might seem wrong. She highly values Harry’s wish to return to his former home, given his firmness and tenacity. John remarks that Harry’s tenacity seems to be related to his conviction that promises should be kept. According to John, Harry’s notion of respect does not primarily mean that he wants to choose by himself where to live but that promises which have been made to him are kept. Others recognize this. The participants conclude that showing respect to Harry does not necessarily mean following his wish, but taking into account the importance he attaches to promises.*

*The facilitator summarizes the dialogue and concludes that the main values in the dilemma have been changed. The value that is opposed to happiness and participation is not self-determination but trustworthiness.*

In this step, similarities and differences between the individual considerations are examined. Sometimes, two participants make a different choice in the dilemma based on the same value. On the other hand, participants may choose the same option in the dilemma based on different values or norms. Identifying similarities and differences may lead to a better understanding of one another and a better insight in what is important in the specific case. Thus, the participants reflect on their own values and learn to see the relevance of other positions. In dialogue, they may reach a new and richer view of the situation. A dialogue is distinguished from a discussion. In a discussion, the participants try to persuade each other that their own position is superior. In a dialogue the participants focus on understanding and examining each other’s viewpoint. A dialogue requires a critical yet constructive attitude of listening and asking questions.

### Step 9. Conclusion

*The participants go into the consequences of the outcome of the previous deliberation, which resulted in the insight that Harry’s wish to return is not induced by his attachment to his old home but by his conviction that promises should be respected. They conclude that this is not a good basis for organizing a move. Thus, the decision is to make Harry stay where he lives now. It is also decided that it is necessary to do justice to the importance which Harry attaches to promises. The team leader proposes to ask the personal caregiver of Harry in his former home, who made the promise, to discuss this with Harry. She expects Harry will accept an explanation by the former caregiver that the promise was premature. If this will not work out in a satisfactory way, a new MCD meeting will be arranged.*

In this step, the participants are invited to sum up conclusions and make a plan for action. The facilitator returns to the moral question which was formulated at the start of the MCD, and asks the group to make explicit the insights which have been reached. These insights can relate to the issue at stake, to the joint reflection process, or to some basic key principles that can be a starting point for a similar case in the future or a corner stone for developing policy or guidelines concerning the more abstract more issue that lies behind this specific case. Reaching consensus is not necessary; the conclusion can also be that there is a plurality of ideas which lead to questions what this plurality means for daily practice and how to deal with it. In case one idea or participant is dominant, the facilitator might ask questions to encourage critical reflection among the participants. This may open the dialogue again and lead to new ideas and conclusions. A critical and Socratic attitude of the MCD facilitator is crucial here. Sometimes, conclusions should be understood as preliminary conclusions because a referral to an external expert or another perspective is needed after the MCD sessions. In case of limited time, this step can be shortened to a brief inventory of the conclusions of the participants or a summary by the facilitator.

### Step 10. Evaluation

*The facilitator evaluates the MCD with the participants. What are the results of the case discussion and the MCD? How was the process experienced? The attendees indicate they acquired a better insight in the dilemma and a better understanding how to take Harry seriously without acting immediately in order to meet his wish of returning to his old home. The family feels satisfied because their worries have been taken seriously. All participants mention they experienced the conversation as open and constructive.*

Evaluation is important in order to learn from positive and negative learning experiences regarding the process and the result of the moral deliberation. This may also lead to changes concerning the skills, attitudes and procedure next time, taking into account limitations experienced.

## Results and Discussion

The dilemma method is a specific conversation method for MCD, which fosters reflection of and dialogue between professionals on a concrete case in their own practice. The teaching of bioethics through MCD in general, and the dilemma method in particular, takes seriously the actual moral concerns of professionals in practice, as their own cases and experiences are the point of departure. It makes professionals aware of their presuppositions and their reasoning skills and attitude. It also broadens their thinking by focusing on a variety of perspectives and exchange of views. In the following we will discuss the specific characteristics of the learning process, experienced by the participants, and go into the role of the facilitator as a teacher.

### The moral learning process of the participants

MCD in general, and the dilemma method in particular, does not focus on raising knowledge of ethical concepts, theories and argumentations, nor on reaching consensus in argumentation or decision-making, but on fostering practical rationality and moral competence. This way of teaching ethics fits in with competency-based learning, which is currently emphasized in medical teaching. It is in line with the CanMEDS approach [31], which focuses on competences of the physician. One of the competences distinguished in the CanMEDS approach is professionalism. MCD may enhance professionalism; participants point out that MCD not only makes them think and act differently, but also makes them better professionals [8]. MCD contributes to the development of reflective professionals, who possess the deliberate and moral skills needed to have a constructive dialogue and to justify their actions [32].

In MCD, participants develop specific moral competences. These include: growing awareness of their behaviour and thinking, listening critically and sincerely, postponing moral judgements and an awareness of perspectives of others. Participants indicate that the method helps them to gain a better insight in the moral issues in a case [7, 8, 32]. The method results in actions that are rooted in a) their own convictions and reasoning (i.e. conclusions do not come from theory or experts), and b) the concrete context in which the moral question emerged. The close connection between the moral problem and the process of reasoning and finding a solution within one and the same context makes MCD an effective learning method. A crucial element of teaching bioethics in MCD is fostering an exchange of perspectives through dialogue. MCD makes professionals aware of their own presuppositions and thinking process, and broadens their views. Evaluation research shows that participants experience this as a central feature of moral learning [29].

By defining ethical issues in terms of two mutually excluding options, the dilemma method makes the moral dimension of a case concrete. The participants are made aware that ethical issues are practical: a moral decision makes a difference in practice. The formulation of a dilemma also makes clear that moral decisions entail costs. If one decides for option A, one cannot realize the values that underlie option B. Through MCD participants learn that dealing with moral issues comes at a cost. Moral life is inherently tragic [33].

The dilemma method focuses on developing moral competence, which includes both knowledge and skills. The method creates awareness of both the existence and status of values in moral life. Moreover, it promotes reflection and deliberation on the concrete meaning and implications of specific concepts and values. In the case example, the value of respect for autonomy was investigated by the group. Whereas, at first, respect for autonomy was interpreted by most of the participants in terms of following the wish of Harry and enabling him to return to his former residence, it later became clear that, for Harry, respect for autonomy meant that a promise made to him should be taken seriously. Respect thus would entail showing that Harry could trust that former agreements would not be ended one-sidedly. This resulted in the conclusion that respect for Harry might take the form of discussing the limitations of the promise with him. Thus, the group developed the insight that respecting autonomy does not simply mean following the other’s wishes, but creating a relationship of trust, in which the other experiences being treated and respected as a person.

### The facilitator as teacher

Facilitating MCD, applying the dilemma method, is different from giving a lecture on ethics or explaining a moral concept or argumentation. The role of the facilitator in MCD is not to elucidate theories from textbooks, but to help participants to reflect on their own moral experiences and reasoning, make explicit the values involved and become open for other values and perspectives. Asking questions is a central feature in MCD. The facilitator should be a Socratic teacher who possesses the art of ‘maieutics’ [25, 26]. He or she should be a role-model in the Socratic attitude and encourage the participants to question rather than to argue. Through questioning, MCD participants develop a more active learning style. The facilitator has a substantial and active role in helping participants to deepen their moral point of view. In order to acquire skills, knowledge, attitude and a specific view on ethics, a facilitator needs a solid training [34, 35].

The facilitator should primarily foster the process of reflection and dialogue in the group. This, however, requires insight in ethical issues and concepts. The facilitator should be able to explain to the group what distinguishes an ethical question from a practical one, and to elucidate the nature of a moral dilemma. He or she should be able to explain what values and norms are, how they are related, and help the group to formulate the values and norms of various stakeholders in the case. The facilitator should be able to stimulate and support the group in investigating specific values. Knowledge of ethical theories and concepts may be helpful in this respect. Yet, the facilitator should be careful not to apply theoretical knowledge too quickly, and be open to possible new interpretations of concepts which differ from those in the literature. Thus, the facilitator should not simply write down a concept mentioned by a participant, for example ‘respect for autonomy’, but help him or her in investigating its meaning in the concrete situation. This may then result in a new and richer view of the concept, as we saw in the dialogue on Harry’s views on the relationship between respect for autonomy and taking seriously former promises.

The facilitator should also have knowledge of the theoretical background of the method and the various steps involved, but most of all he or she should be able to apply the method in a context-sensitive way. The facilitator should foster a joint inquiry and dialogue rather than following mechanically the method step by step. The steps within the method should support the process and the moral inquiry. That means the MCD facilitator should focus on the content of the deliberation. The facilitator, like the participants, should listen to what is being said – and sometimes not being said. Eventually the method should support the process of getting insight into the issue at stake. Method is not important in itself, but only as a means to get insight into what Gadamer calls truth: the method should lead to a joint learning process and broadening of horizon, resulting in an increased insight into what really matters in the case and what is right to do [22, 24].

## Conclusion

Bioethics education in academic programs for medical students or nurses often aims at knowledge transfer and deductively applying ethical principles or theoretical frameworks to textbook cases. MCD is a specific form of bio-ethics education in the context of clinical practice, which focuses on real cases and moral issues that are actually experienced by health care professionals. The approach to MCD presented in this paper is based on hermeneutic ethics, practical rationality and a Socratic epistemology. MCD follows an inductive learning approach through a dialogical moral inquiry in which participants develop not only knowledge but also skills, attitude and character. The dilemma method—a specific conversation method used for MCD—and its underlying view on teaching ethics are useful for supporting health care professionals in and teaching them how to reflect on their own moral issues in practice. MCD participants report that they learn to recognize the moral dimension of daily practice and feel more able to distinguish various perspectives and reason in a systematic manner. The facilitator as teacher focuses not on explaining moral theories and concepts, but helps the participants to reflect on their experiences, presuppositions and reasoning through a dialogical moral inquiry with others.

## Abbreviations

*CES* Clinical Ethics Support; *MCD* Moral Case Deliberation
